# Introductory to the Bureau of Materia Medica

**Published:** 1896-09

**Authors:** W. A. Yingling

**Affiliations:** Chairman, Emporia, Kan.


### INTRODUCTORY TO THE BUREAU OF MATERIA MEDICA.

KANSAS STATE MEDICAL SOCIETY, 1896.

W. A. YINGLING, M. D., CHAIRMAN, EMPORIA, KAN.

Possibly no part of the book world has as many obsolete and valueless volumes, save as curios, as the allopathic. On the other hand, no part of this great book public has as many valuable books, usable books, really indispensable books written two or more decades ago, as the homœopathic. This fact alone should plainly indicate which of the two schools is "regular" and based upon unchanging law. The most valuable books of the present day to the homœopathic student, those that form the basis of all others, save only the newer pathopoieses of remedies, are those penned by Hahnemann and his coadjutors. When the author of to-day seeks the fountain of homœopathic authority, either for philosophy or the indications for remedies, he at once turns to the writings of Hahnemann and his recognized colaborers. The reason for this is not sentiment, but necessity. Law is unchanging, stable, and permanent. That which was a fact yesterday is a fact to-day, and will be a fact to-morrow. Time makes no changes in the warp and woof of Truth. In this the homœopathic has the greatest advantage over all other systems of medicine. The homœopathic indications of drugs never change, never become obsolete. There is no unlearning the lessons of yesterday, no need of forgetting the teachings of former years, unless, alas! the knowledge was obtained at certain so-called homœopathic colleges. We may and do learn more of remedies by experience, but the pathopoieses of the remedies never change. The indications for Belladonna given by Hahnemann are as valuable and reliable to-day as they were when penned by the hand of that true philosopher and teacher. How different in any other department of science or philosophy! New discoveries make inroads into old beliefs, or the change in specific knowledge based upon newer facts changes the status of usable material. The allopath is the snake of medicine; he must change his coat every season. Yet there are some who pick up the cast-off garments in hopes of finding an easy way to cure the sick. The homœopathic materia medica is like the laws of the Medes and Persians, ever being added to, but never changing. The old books are the good books, and no one will willingly part with them, especially no one who has learned to rely on the Law of Similars in the cure of disease.

The Law of Potentiation is next in importance to the Law of the Similars. The exhibition of the miraculous power of many seemingly inert substances proves this postulate to be true. The question is not of high or low potencies, but of potentiation in the development or the liberation of the hidden curative power of substances and drugs. But for the recognition of this law our materia medica would be emasculated of some of the most valuable and indispensable remedies. Like the long-hidden X or Rœntgen ray, with its power of penetration and effect upon the sensitive plate, the potentiation of drugs, with its equally penetrating and subtle power in bringing forth the curative properties of drugs, was hidden in the vast arcanum of nature till the mind of him whom we all love to honor was divinely led to its discovery. Like the X ray, potentiation may be incomprehensible, beyond the ken of man's mind, yet the results are as tangible and as wonderful. Were we constrained to reject all we do not comprehend, the bulk of our boasted knowledge and accomplishment would be relegated to the domain of fairyland, where the elfish glee would crimson the cheek of man's science and put to shame his boasted advancement. Man is an *a posteriori* being; he must accept the results of the law he finds strewn along his pathway, and from them go backward to discover the cause. His mind reasons from effect to cause in his discoveries; he is limited to the tangible effects of law in his acquisition of higher knowledge. Whilst he is groping along in the darkness of his ignorance some happy chance opens the doors and invites him to enter the inviting field of the unknown. The wise and sincere holds in abeyance his judgment till nature plainly speaks the truth, whereas the simple and self-conceited rejects without a hearing and denies that which his simple and uneducated mind cannot comprehend.

There is a "something" in the substance behind the material, or in the material, that potentiation liberates and sets free. It cannot be that the comminution of the particles of the substance gives the added potency, else pulverization would be equal in its power to potentiation. Potentiation contains all that comminution can imply, but it goes beyond the domain of comminution, even where the microscope cannot follow. We are taught that the divisibility of matter is illimitable, and that matter cannot be destroyed; but may it not be possible that there is a point where the breaking-up of the atoms liberates that "something" which distinguishes the one substance in its being and effect from every other substance—its very life? The fact that potentiation is an unknown factor, or is novel, no more proves it untenable than the same fact proves the unknown quantity or X ray a myth. We accept the X ray (so-called because unknown) upon its results. The results of potentiation are just as tangible to those who have sincerely investigated it, and hence must be accepted by all true scientists. It seems strange that so many are willing to accept the assertion of several scientists, without even a passing doubt, as to the wonderful powers of the X ray, yet reject the positive experience and declaration of a very large number of equally veracious men as to the wonderful effect of potentiation.

There is a tendency with a part of the profession to reject the nosodes. They call them "nasty products" of disease, implying that the "nastiness" is administered, and yet forgetting that potentiation removes all the material as they claim. If the material is wholly lost in potentiation, then surely the "nastiness" is fully removed, and the nosode becomes as palatable as the choicest remedy. Some who lustily reject the homœopathic nosode are eager to accept isopathic products, and insist upon the injection into the system of Pyoid and Pyogenic virus from the lower animals in the form of Vaccine, Antitoxine, Hydrophobine, and so forth. Surely pus or other products from the animal is as "nasty" as the same from man, and it is just as "nasty" to inject the product into the system by the medium of the skin as by the usual channel of the mouth. The snake poison taken by the mouth is innocuous and innocent, but when injected through the skin becomes venomous and a destroying poison. Who can assert positively that the animal product does not act in the same way? Is it not less harmful or injurious to give the nosode as other medicine is given than to inject the serum hypodermically? Then, again, the injection of the serum is palpably isopathic, whereas by potentiation the diseased product is so changed as to remove all "nastiness," and transform it into a harmless remedy with wonderful curative properties.

The scientific world to-day is very blunderingly stumbling along the path trodden by previous homœopaths. Their rejection of potentiation closes to them the only way by which they could derive any benefit from diseased products. Almost every product being investigated to-day by the so-called scientists has been potentiated and more or less proven or verified by the homœopathic school years ago. These scientists have met with failure, whereas the homœopathic procedure has brought success. Koch's lymph has been relegated to the shelf of unpleasant remembrances, notwithstanding it was hailed as the greatest discovery and highest blessing to suffering man within a lifetime, and the various serums will soon lie beside it, whereas Tuberculinum, Diphtherinum, Anthracinum, Scarlatinum, *et cetera*, are being used more successfully and extensively than ever before.

We can give one rule for the administration of unproven nosodes. To be sure, *it is better to properly prove each one according to the direction of Hahnemann.* That is the only proper way, but it would not be wise, in my estimation, to reject those not thoroughly proven. We should hold on to them as most useful weapons against disease in hope that the time may come when, by verification, clinical experience, or proper proving we may use them not only more intelligently, but more extensively. Nosodes like Tuberculinum, Medorrhinum, Luesinum, Psorinum, Pyrogenum, and a few others, with more or less perfect provings, we can apply to the cure of disease according to the instruction of Hahnemann in *The Organon,* and with marvelous success, by their pathopoieses, pathogensis, and verifications, but with Scarletinum, Morbilinum, Erysipelinum, Verrucinum, and some others, not proven, and with but little or no clinical verification, we must seek another way, especially as there is gold in these mines that should be brought to the surface. In carefully considering this subject in the light of pure Homœopathy, and being opposed to the empirical use of any remedy, whether a nosode or a drug, yet desirous of investigating the homœopathic nosode, I came to the conclusion that *those nosodes having known marked and prominent symptoms,* though few, could be used in cases where *these symptoms are prominent,* but those nosodes without known symptoms could only be used homœopathically in the cure of diseases in one class of cases.

The following case will better illustrate the use of unproven nosodes than any attempted explanation. In this illustration I select a case wherein one of the least known and used nosodes was administered.

Miss McC., æt. forty-eight ; rather dark.

Desires to be relieved of her excessive constipation, which, for the last few weeks, is very alarming to her.

Has taken much physic and Quinine. The tongue is very white and clammy all over, thicker behind. A very bitter, nasty taste. Menses are about normal. Some indefinite leucorrhœa. Has had a number of lymphatic tumors removed from above the clavicle and in the axilla during the last eighteen months, and now others are growing to her alarm. Two years ago she had her right breast entirely removed and a tumor from the right axilla. A yellow dry scab on the cicatrix of the breast. Red eruption over chest, worse when warm. Pain on top of left shoulder, worse on deep breathing. Tremor of hands. Keeps neck warmly wrapped up in flannel; without the wrapping chilliness and pain in the neck. Had nervous prostration years ago. Extremely nervous, much worse since the surgical operation. Chloroform produced palpitation of the heart. When tumors were removed Cocaine was used, which caused dryness of mouth and nervous jactitations. The father had cancer of the lip; about a year after the removal of this supposed cancer, lumps formed on his neck and about it; in a few months the lumps all disappeared under local treatment and "went to his lungs," resulting in his early death.

The patient before us has cold and damp feet up to the ankles. Sweats all over at about four o'clock in the morning when awakening; sweat has been quite warm for several mornings. Thirsty for a quantity of cold water. Dreams usually pleasant, but disagreeable for the past few nights. Feels rested when first arising in the morning, but tired and weary by breakfast time. Face in the morning looks greenish, with purplish spots. Fats disagreeable. Sours aggravate pains in the stomach. Sweets sicken. Painless corns. White spots on nails of hands. Gets out of breath, difficult breathing, on exertion; must move slowly for several years past.

When about twenty her back was very weak, with the sensation as if the vertebra were sliding over each other; applied a strengthening plaster; when she pulled it off by force on account of the itching and burning the skin was broken; following this was great swelling, inflammation, burning, stinging, itching, and redness over the place where the plaster had been. The erysipelatous inflammation extended over back and around the body, involving the eyes, ears, and the face; the eyes were swollen and closed. Various applications were made by the allopathic attendant with no amelioration. A neighbor applied Sulphur, Tar, and Lard with amelioration within an hour, and rapid diminution of the erysipelas. Following this suppression pimples came on the face and elsewhere, and were doctored with sugar of lead; these pimples continued off and on for about three years when they finally disappeared by the continued use of the lead solution. She has been a chronic sufferer ever since she had the erysipelas. A year ago she took for a full year, three times a day, the one-hundredth of a grain of Arsenic, which caused a whizzing in the head, and pain in the occiput; she felt sick after each dose. Three years ago she took daily the Iodides of Potash and Iron for seven months. She also swallowed seven bottles of Ayer's Sarsaparilla, and yet continued the existence that had no charms for her. Six years ago she had the la grippe, the whole neck and jaws were sore, the jaws locked for a time. Jayne's pills and the inevitable Quinine was the treatment. The allopathic doctor, in sheer desperation, advised "some kind" of liniment to be applied to the sensitive glands, and *she* selected the wonder-working Centaur, since which time has come all the glandular enlargement.

She always coughs when lying on her back. She likes company, and to be moving about slowly. When feeling very sick she wants to be alone. She used to bathe every day, but now only once a week, and feels that she should bathe more frequently, as she "smells her body," which is very disagreeable to herself. Appearance somewhat unclean, yet she does not like to wash.

℞ *Erysipelinum*$^{dmm}$ (Swan), one dose dry on the tongue.

The third day after she reports amelioration in every respect, feeling better than for a very long time. Following the exhibition of the nosode an old headache returned for two nights, and every place where an operation had been performed ached and felt sore for a short time. A severe toothache lasted for nearly forty-eight hours, and then passed off without treatment. Sleeps well and feels more refreshed. Hands and feet feel warm, which is a great change for the better. Stool normal, bowels moving each day. This is wonderfully encouraging to her. Ate an apple with pleasure; before she could not eat them. Now has some itching of the shin-bones, worse at night and worse scratching. Looks and acts better in every respect.

I might add that it was not faith nor the effect of taking something that brought this change about. It required several visits to get the history and totality of the case, and during this time she was taking Placebo. Following the administration of the nosode the improvement began, and, according to all precedent, the good work must be attributed to the action of *Erysipelinum.*

As no one remedy was clearly indicated, it would have been but guesswork to have given any particular one. A large number of remedies might have been selected according to the view-point of the prescriber. In this case surgery failed to accomplish anything, and it would have been more than useless to have done more cutting in hope of even palliation. That the nosode given was the true remedy is proven by the results. The reasons for the selection of the nosode were three: First, because there was no clearly indicated remedy. Second, to have prescribed on the last sick condition would only have been to palliate without reason to hope for a permanent cure of the chronic condition, which was the cause of the acute. Third, the whole condition was evidently the result of the suppression of the erysipelas years before, and if that poison were antidoted the effects must be removed.

One of two results will always follow the administration of a nosode under the above conditions—the case will either be cured permanently of all trouble, or it will be so developed as to plainly point to the curative remedy.

I do not advocate the promiscuous and indiscriminate use of any nosode or remedy. *The law governing the selection of a remedy must be followed so far as possible.* When a case is so obscure and complicated as to prevent the application of Hahnemann's directions in the selection of the simillimum, or when several remedies seem nearly as well indicated, and the cause of the disease can be traced to some suppression, or the patient has been unwell since the suppression of some disease without clear indications for one remedy, then the administration of the nosode is not only admissible, but demanded by the nature of the case.

In such a case, if the cause cannot be traced to a suppression of some disease, but can be, by present symptoms or the sickness following the large use of some allopathic nostrum, to a *drug disease,* then the antidotal treatment must be utilized. That the very high potency will antidote the *poisonous effects* of the same crude drug is true, is now beyond question to my mind, for I have antidoted the results of allopathic drugging with curative effect, and other credible practitioners have had the same experience. Experience speaks louder than opinion every time.

Homœopathy is a science, and the only proper way to become proficient in its application to the cure of the sick is to study and master its philosophy. A man without a streak of philosophy in his make-up has no business in the homœopathic ranks. The true homœopath must be a philosopher, or, at least, be able to view the subject philosophically. The successful homœopath must be able to view and grasp principles, to discriminate, to weigh evidence, to see the relation of things and decide upon cold facts without bias or prejudice. Without this philosophical mind he will either become a bungling pathological prescriber or a mere symptom-coverer, neither of which is a homœopath in the true sense of the term. The true homœopath is not tied down to biased isms or preconceived opinions, but to principle and rational experience. He must have a clear head, an unclouded mind, a discriminating judgment, and a philosophical memory; that is, a memory that retains principles and conditions, the genius of drugs, rather than isolated symptoms and crude pathopoieses.

The homœopathic materia medica is the great spook to frighten the pathological prescriber, but like Banquo's ghost, it will never down; it must ever be the first desideratum to the follower of Hahnemann, if he desires success. It is not so formidable when properly considered. To such a mind as we have ventured to suggest as essential to the true homœopath, it becomes the pleasant pastures and sparkling rills beside which he can feast in comfort and ecstasy. Herein he rambles to the satisfaction of his soul and quaffs the cool and limpid waters of a perpetual delight. It is his meat and drink, and will become his success and fortune.

The best prescribers are not those who can quote the most materia medica, but they who can apply the most. We have too many parrots who glibly repeat what they have learned by dint of hard and persistent application, yet without intelligence and mental grasp. The true homœopath tries rather to comprehend, to mentally grasp the ideas conveyed, than to remember the language, and then when he sees the picture of the disease before him on the bed, or in the office chair, the association of ideas, in accordance with true memory, brings before the mind that other picture contained in the drug. It is the pathogenesis developing the photo of the pathopoiesis. One hour of comprehensive study is equal to a whole day's memorizing. That which is memorized will gradually fade away and become so indistinct as to be practically useless, but the comprehensive assimilation of ideas and facts will become an indelible picture ever responding to the magic touch of the clinical button.

